# The biochemistry underpinning industrial seed technology and mechanical processing of sugar beet

**DOI:** 10.1007/s00425-019-03257-5

**Published:** 2019-08-14

**Authors:** Michael Ignatz, James E. Hourston, Veronika Turečková, Miroslav Strnad, Juliane Meinhard, Uwe Fischer, Tina Steinbrecher, Gerhard Leubner-Metzger

**Affiliations:** 1 Department of Biological Sciences, Royal Holloway University of London, Egham, Surrey TW20 0EX UK; 2 KWS SAAT SE & Co. KGaA, Grimsehlstr. 31, 37555 Einbeck, Germany; 3 Laboratory of Growth Regulators, Palacký University and Institute of Experimental Botany, Czech Academy of Sciences, 78371 Olomouc, Czech Republic

**Keywords:** Abscisic acid (ABA), Germination inhibitors, Pericarp (fruit coat), Polishing and washing, Seed processing, Seed technology, Sugar beet (*Beta vulgaris* subsp. *vulgaris*)

## Abstract

**Main conclusion:**

Seed-processing technologies such as polishing and washing enhance crop seed quality by limited removal of the outer layers and by leaching. Combined, this removes chemical compounds that inhibit germination.

**Abstract:**

Industrial processing to deliver high-quality commercial seed includes removing chemical inhibitors of germination, and is essential to produce fresh sprouts, achieve vigorous crop establishment, and high yield potential in the field. Sugar beet (*Beta vulgaris* subsp. *vulgaris* var. *altissima* Doell.), the main sugar source of the temperate agricultural zone, routinely undergoes several processing steps during seed production to improve germination performance and seedling growth. Germination assays and seedling phenotyping was carried out on unprocessed, and processed (polished and washed) sugar beet fruits. Pericarp-derived solutes, known to inhibit germination, were tested in germination assays and their osmolality and conductivity assessed (ions). Abscisic acid (ABA) and ABA metabolites were quantified in both the true seed and pericarp tissue using UPLC-ESI(+)-MS/MS. Physical changes in the pericarp structures were assessed using scanning electron microscopy (SEM). We found that polishing and washing of the sugar beet fruits both had a positive effect on germination performance and seedling phenotype, and when combined, this positive effect was stronger. The mechanical action of polishing removed the outer pericarp (fruit coat) tissue (parenchyma), leaving the inner tissue (sclerenchyma) unaltered, as revealed by SEM. Polishing as well as washing removed germination inhibitors from the pericarp, specifically, ABA, ABA metabolites, and ions. Understanding the biochemistry underpinning the effectiveness of these processing treatments is key to driving further innovations in commercial seed quality.

## Introduction

After the harvest of dry fruits and seeds, innovative industrial technologies such as cleaning, sizing, washing, drying, dehulling, polishing, priming, coating, or pelleting are applied (Sliwinska et al. 1999; Kockelmann and Meyer 2006; Sharma et al. 2009; Pedrini et al. 2017; Steinbrecher and Leubner-Metzger 2017; Chomontowski et al. 2019). Processing technologies are also applied to dry seeds and fruits for the production of fresh sprouts of *Beta vulgaris* subsp. *rubra* L. (red beet) and for the production of vigorous seedlings to support primary crop production of *Beta vulgaris* subsp. *vulgaris* var. *altissima* Doell. (sugar beet) in the field (Dewar et al. 1998; Kockelmann and Meyer 2006; Latorre et al. 2012; Metzner et al. 2014; Blunk et al. 2017). For commercial seed production, washing, dehulling, and polishing technologies are used to improve the germination performance by removing physical (“hardness”) and chemical (germination inhibitors such as abscisic acid and its metabolites) constraints conferred by the seed and fruit coats. Modifying the pericarp (fruit coat) is also used to eventually reduce infestation with pathogens which are localised in the pericarp (Fukui 1994).

**Fig. 1:**
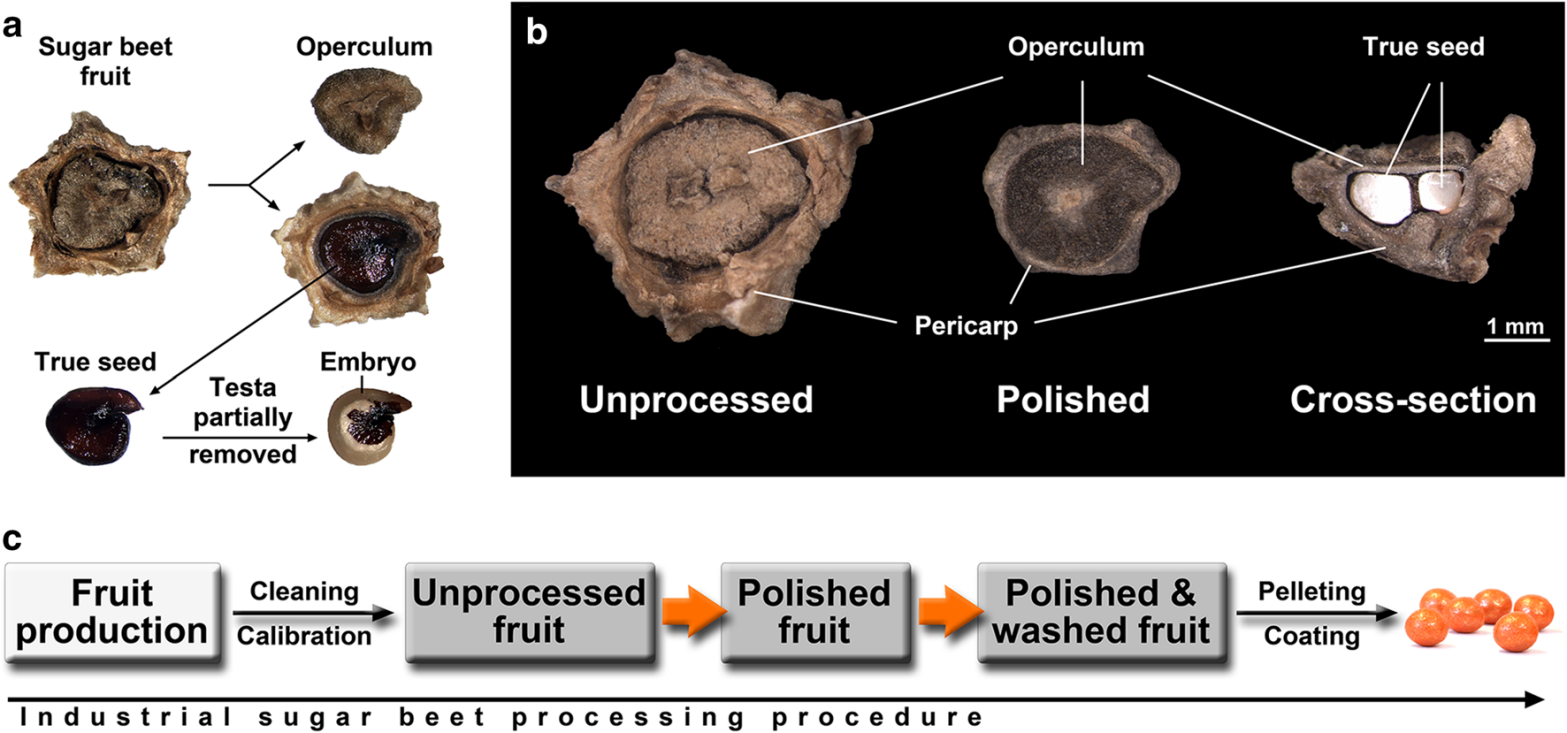
**a** Mature fruits and seeds of *Beta vulgaris* L. The sugar beet seed is enclosed by a fruit coat (pericarp) which possesses a lid-like structure (operculum). The seed consists of an embryo and seed covering layers (endosperm and testa). The curved embryo surrounds a starch storage tissue (perisperm) of maternal origin. **b** Overview of an unprocessed and processed (polished) sugar beet (*Beta vulgaris*) fruit (left and middle). A manually fractured sugar beet fruit is shown on the right with a side on view, revealing the true seed inside the pericarp. **c** Scheme depicts the industrial sugar beet processing process (from harvested fruit to commercially sold pelleted fruit), including the polishing and washing step

Sugar beet is a plant in the Amaranthaceae family that is a crop of high global importance, as it is the major source of sugar in temperate zones. It provides up to 30% of the world’s annual sugar production (Dohm et al. 2013; Frese 2010; Blunk et al. 2018). In sugar beet, red beet, spinach, and other Amaranthaceae food and feed crops, the harvested “seed” is botanically a fruit, consisting of the true seed surrounded by the fruit coat (pericarp), derived from the carpels, and the incorporation of other parts of the flower (Artschwager 1927; Hermann et al. 2007; Lukaszewska and Sliwinska 2007; Deleuran et al. 2013). The true seed is composed of a fragile and brittle seed coat (testa) covering the coiled embryo which is curled around the central perisperm, a starchy storage tissue descended from the nucellus (Fig. 1a). The germination of sugar beet is largely controlled by the pericarp, which functions as both a physiochemical barrier and a reservoir of inhibitory substances. The complex nature of this inhibition has been debated at length by researchers for several decades (De Kock and Hunter 1950; Snyder 1965; Chetram and Heydecker 1967; Coumans et al. 1976; Junttila 1976; Chiji et al. 1980; Santos and Pereira 1989; Taylor et al. 2003; Hermann et al. 2007; Abts et al. 2014, 2015; Blunk et al. 2017).

Industrial sugar beet seed technology and mechanical processing can have a major impact on the pericarp, but information on the biochemical mechanisms underpinning these processes is sparse. The pericarp of a harvested, unprocessed dry sugar beet fruit consists of several cell layers, with the two most prominent: an inner layer of thick-walled sclerenchyma cells and a porous outer layer of large parenchyma cells (Figs. 1b, 7d this work; Artschwager 1927; Orzeszko-Rywka and Podlaski 2003; Hermann et al. 2007; Lukaszewska and Sliwinska 2007). The operculum (fruit cap) is a lid-like structure on the upper part of the pericarp (Fig. 1). A basal pore is located on the bottom part of pericarp. It is a pore-like pericarp structure comprised of loose cells. The operculum and basal pore have both been proposed as major entry points for water and oxygen during germination (Richard et al. 1989; Santos and Pereira 1989).

In the field, commercial sugar beet production is a labour-intensive process requiring a large cultivated area as well as a long and stable vegetative period (Kockelmann et al. 2010). Farmers demand “seed” of the highest quality and germination performance. To meet this demand, expensive processing and seed technologies are applied that contribute to sugar beet fruits’ high commercial value. Various enhancement treatments are applied to the fruits during the production and processing pipeline to further improve the quality characteristics for optimal plant establishment (Draycott 2006; Kockelmann and Meyer 2006; Kockelmann et al. 2010; Lukaszewska et al. 2012). Such processing treatments often have a positive impact on the germination performance of sugar beet by maximising germination speed, as well as capacity and uniformity of germination and seedling establishment in the field.

In this work, we focus on two processing treatments, polishing and washing. Polishing is used to remove parts of the pericarp tissue, resulting in a fruit size and shape which is suitable, after assessment for any damage to the seed within (Salimi and Boelt 2019), for pelleting (Draycott 2006; Halmer 2008; Kockelmann et al. 2010; Pedrini et al. 2017). Partial removal of the pericarp by polishing also leads to an improved germination performance. Washing applied after the polishing is often a prerequisite for pelleting methods. It improves seed quality, likely by flushing out germination inhibitors from the remaining pericarp (Draycott 2006; Kockelmann et al. 2010). Polishing and washing are two common treatments used during seed production and both are known to have a positive effect on the germination performance (Orzeszko-Rywka and Podlaski 2003; Tohidloo et al. 2015). Although these treatments are often applied during sugar beet seed production, little is known as to how these treatments affect the biochemical properties of the pericarp. Do they remove the physicochemical constraints, including inhibitors, to achieve improved germination performance? Which of the changes in fruit properties determine the effectiveness of the applied method?

Our work aims to investigate the physiological and biochemical basis of the polishing and washing processing treatments in comparison with unprocessed sugar beet fruits. We analyse how these technologies affect the germination performance, the contents of pericarp inhibitors, ABA (Hermann et al. 2007), and ions (Snyder 1965), in connection with morphological changes in the pericarp caused by polishing and washing.

## Materials and methods

### Plant materials

Fruits of sugar beet (*Beta vulgaris* subsp. *vulgaris* var. *altissima* Doell.), seed lots A and B were obtained from KWS SAAT SE & Co. KGaA, Einbeck, Germany (with lots A and B corresponding to KWS595 and KWS253, respectively). Lots A and B are different genotypes which share no common element in the parent lines. Lot A was grown in France, while lot B was produced in Italy. Both seed lots were grown and produced in the summer of 2014. As is common in the harvesting process, mature plants were cut and left on swath for drying in the sun. After threshing of plants, seeds were further dried to < 10% moisture content if necessary. Harvested and dried seeds were subjected to a (pre-)cleaning process and calibrated into different size classes (Kockelmann and Meyer 2006; Kockelmann et al. 2010). Subsequently, batches of size-calibrated seeds were polished, making use of a hulling device specifically modified for sugar beet, by bringing seeds into contact with an abrasive material. The process was adjusted specifically for both genotypes to achieve good polishing effects (reduced pericarp; round shape; useable caliber size) but to avoid concurrently any damage (chipped or de-capped seed) negatively affecting germination quality (Klitgard 1978; Kockelmann et al. 2010; Salimi and Boelt 2019). Furthermore, the removal of pericarp material reduces the amount of pericarp-inherent germination inhibitors, an effect which is further intensified by a consecutive washing procedure (Longden 1974). Samples for experimental comparisons are defined as ‘unprocessed’ (no polishing, no washing), ‘polished’ (no washing) and ‘polished + washed’. The 1000 fruit weight of the differently processed fruits was determined according to ISTA (International Seed Testing Association) standard protocols, measured by weighing 8 replicates of 100 fruits and extrapolating a mean weight of 1000 seeds. The reduction in cross-sectional area was measured with the software tool ‘Fiji’ (Schindelin et al. 2012) for at least 190 individual fruit scans. The moisture content, expressed as percentage, was determined by measuring the reduction of fruit weight after drying for 8 h at 105 °C and then dividing by the dry weight, for 5 replicates of 15 fruits each and calculating a mean. Eight hours was selected as the drying time, as there was no significant, measurable decrease in mass beyond this timepoint.

### Germination assays

Germination assays were conducted in darkness at 10 °C (incubator MIR-254-PE, Panasonic, Osaka, Japan). The conditions of 10 °C and darkness are a commonly used industry standard which represent a sub-optimal condition that has been found to better correlate with field emergence than tests carried out at more optimal temperatures (Draycott 2006; Chomontowski et al. 2019). Sugar beet fruits were incubated in white plastic boxes (180 × 135 × 65 mm) with transparent lids containing a sheet of filter paper and a pleated filter paper (Hahnemuehle, Dassel, Germany) which act to separate and provide universal water uptake for the fruits. Four replicates (boxes), each containing 50 sugar beet fruits and 30 ml of distilled water (dH_2_0), were used to ascertain a baseline of germination for each of the sugar beet lots (A and B). Germination in this assay and in all subsequent assays was defined as the radicle (root tip) protruding through and beyond the margin of the operculum. These germination assays were used to compare the effects of the different treatments; unprocessed, polished, polished + washed.

### Seedling phenotype assessment

The seedling phenotype was assessed in both lots (A and B) by incubating sugar beet fruits, as described in “Germination assays”. Seedlings were observed after 13 days at 10 °C followed by 3 days at 20 °C, both under dark conditions. Normal seedlings are defined as seedlings that exhibit a root without discolouration, a straight hypocotyl, and both cotyledons present and open. Photographs of example normal/anormal seedlings were taken.

### Germination assays with pericarp washwater

Pericarp washwater was produced by separating and crushing pericarps from unprocessed sugar beet fruits (lots A and B) with a mortar and pestle and then suspending the crushed pericarp in dH_2_O at a ratio of 1:10 (w/w) in a 50 ml tube (Sarstedt AG & Co. KG, Nümbrecht, Germany). The pericarp-water suspension was then shaken on a laboratory rotator model G2 (New Brunswick Scientific, USA) for 8 h at 200 rpm. Pericarp material was then removed by centrifugation (Eppendorf 5430R, Hamburg, Germany) for 5 min at 2500 rpm (734*g*). Washwater from lots A and B was kept separate. Three treatments were prepared to act as a comparison to the two wash-water treatments. These treatments were either; 3 ml 0.1 mM *cis*-S(+)-abscisic acid (ABA, Duchefa, Haarlem, The Netherlands) solution, 3 ml 100 mM NaCl, or 3 ml of dH_2_O. Wash-water osmolality was analyzed using an osmometer type OM 806 (Loeser Messtechnik, Berlin, Germany) and conductivity was measured using a Jenway conductivity meter 4510 (Cole-Parmer, St Neots, UK).

To access the effects of these treatments, germination assays were performed in 9 cm Petri dishes lined with two ∅85 mm filter papers (MN 713, Macherey–Nagel, Düren, Germany) with 3 ml of pericarp washwater, 0.1 mM ABA, 100 mM NaCl, or dH_2_O (control), respectively. Three Petri dishes containing 25 fruits were prepared for each treatment and were incubated at 10 °C in darkness (Panasonic MIR-254-PE). All fruits were placed with the operculum facing upwards (and basal pore-facing downwards), as the orientation can impact germination (Santos and Pereira 1989).

### ABA and ABA–metabolite extraction and quantification

Pericarps and separated true seeds, from all processing treatments, were snap frozen in liquid nitrogen, ground with pestle and mortar, and then freeze dried. The ABA and ABA–metabolite contents were measured in 5 × 20 mg powdered samples, as described in Voegele et al. (2012). Each replicate consisted of 50 individual samples (pericarps/true seeds).

Internal standard mixtures, containing 20 pmol of each of (−)-7′,7′,7′-^2^H_3_-phaseic acid; (−)-7′,7′,7′-^2^H_3_-dihydrophaseic acid; (−)-8′,8′,8′-^2^H_3_-neophaseic acid; (−)-5,8′,8′,8′-^2^H_4_-7′-OH-ABA (National Research Council, Saskatoon, Canada); (+)-4,5,8′,8′,8′-^2^H_5_-ABAGE and (+)-3′,5′,5′,7′,7′,7′-^2^H_6_-ABA (Olchemim) and 1 ml cold methanol/water/acetic acid (10/89/1, by vol.) were added to the samples. After 1 h of shaking in the dark at 4 °C, the homogenates were centrifuged (36,670*g*, 5 min, 4 °C) and the pellets were then re-extracted in 0.5 ml extraction solvent for 30 min. The combined extracts were purified by solid-phase extraction on Oasis^®^ HLB cartridges (60 mg, 3 ml, Waters, Milford, MA, USA) and then evaporated to dryness in a Speed-Vac (UniEquip). Subsequently, the evaporated samples were methylated, purified by ABA-specific immunoaffinity extraction (Hradecká et al. 2007) and analyzed by ultra-performance liquid chromatography–electrospray ionization tandem mass spectrometry [UPLC-ESI(+)-MS/MS] (Turečková et al. 2009).

### Pericarp total solute content examination by osmolality and conductivity

Powdered sugar beet pericarp samples were prepared in 5 × 25 mg (following the method outlined in the last preceding section) and suspended in 500 µl dH_2_O by heating and shaking (65 °C, Eppendorf Thermomixer 5437) for 30 min. After centrifugation (10 min, 13,000*g*, Centrifuge 5430R, Eppendorf), the clear supernatant was retained for analyses. Osmolality and conductivity were measured as described above.

### Scanning electron microscopy

Electron microscopy of the surface of the pericarp and fruits after a longitudinal fracture was carried out to compare the structure when it had undergone different processing: unprocessed, polished only, polished, and washed. Dry fruits of lot B (whole or fractured) were mounted on 12.5 mm Cambridge aluminium specimen stubs, using conductive putty (Lennox Educational, Dublin, Ireland). Samples were sputter-coated with a 40 nm gold layer using a Polaron SEM Coating Unit E5100 (Bio-Rad Microscience Division). Pericarps were examined using SEM (Hitachi S-3000N, Tokio, Japan, acceleration voltage 20 kV), with images subsequently contrast adjusted in Adobe Photoshop CS5.

### Graphics and data analysis

Graphics and curve fits using a hill function for germination data were created using GraphPad Prism (version 7, GraphPad Software, San Diego, CA, USA). For germination *T*_50%_ (the time required for a population to complete 50% germination), anormal seedling phenotype, phytohormone, and conductivity/osmolality data ANOVAs followed by Tukey’s HSD post hoc tests were performed using R (version 3.6.0). Germination *T*_50%_ values were calculated using Germinator (version 2.01; Joosen et al. 2010). Where results of the post hoc tests are represented on the graphs, different letters represent significant differences with a confidence interval of 95%. All figures show means and standard error.

## Results

### Comparative analysis of polishing and washing on sugar beet fruit germination and seedling phenotype

The industrial processing of the raw sugar beet fruit (‘unprocessed’) by polishing (‘polished’) removed a considerable amount of the outer pericarp tissue, as shown in Fig. 1b. The quantification of this showed that the polishing reduced the fruit size (cross-sectional area) by ~ 44% and 49% for lot A and lot B, respectively, and reduced fruit mass by 27% and 23%, respectively (Table 1). The ‘polished + washed’ fruits were dried back after the washing step. Due to this, the ‘polished’ and ‘polished + washed’ fruits did not differ significantly in size and mass, and all fruits had a similar moisture content of ~ 8.5% (Table 1). To assess how the polishing and washing affected the fruits of the two lots, we conducted germination assays.

**Table 1:** Summary data (mean ± SE) of *Beta vulgaris* L. fruit mass and moisture content

Seed technology	1000 fruit weight^a^ (g)	Reduction in pericarp mass after polishing^a^ (%)	Reduction in size (cross-sectional area)^b^ (%)	Moisture content^c^ (% DW)
Lot A				
Unprocessed	18.1 ± 0.3	n/a	n/a	8.7 ± 0.2
Polished	13.2 ± 0.1	35.3	44.4	8.6 ± 0.1
Polished + washed	13.1 ± 0.1	36.2	42.7	8.3 ± 0.1
Lot B				
Unprocessed	15.9 ± 0.2	n/a	n/a	8.5 ± 0.1
Polished	12.3 ± 0.1	31.1	48.6	8.5 ± 0.3
Polished + washed	12.0 ± 0.1	33.8	48.7	8.5 ± 0.2

^a^*n* = 8 × 100 fruits

^b^*n *= 190 fruits

^c^*n *= 5 × 15 fruits

**Fig. 2:**
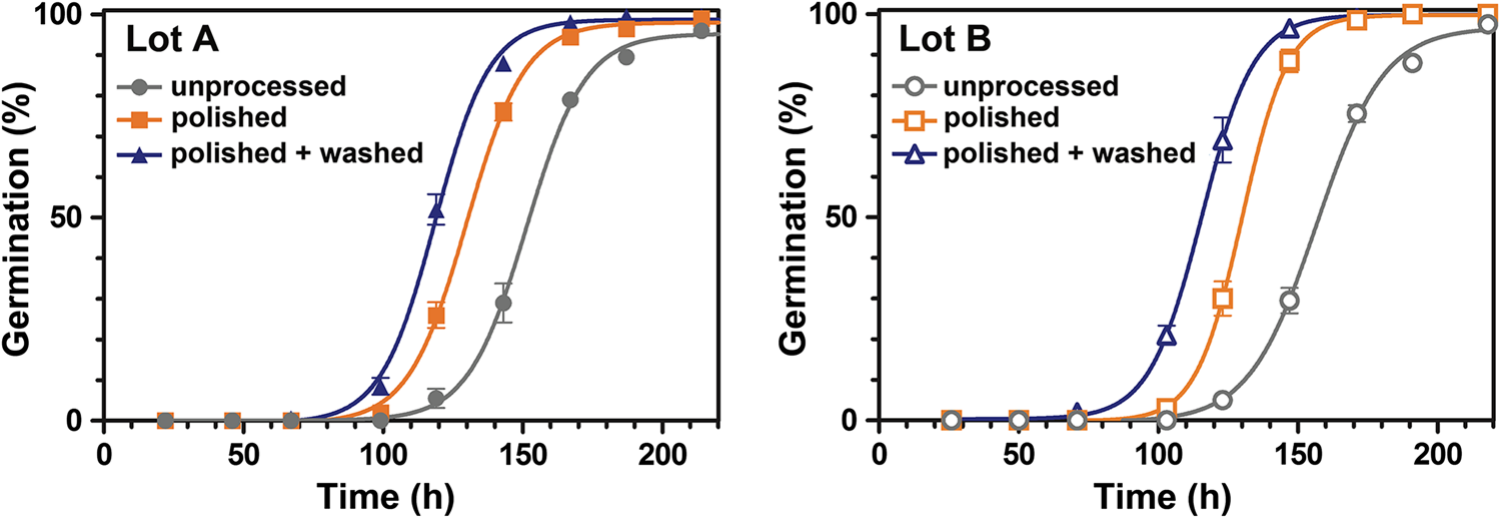
**a** Germination kinetics for sugar beet fruits imbibed at 10 °C in darkness, for lots A and B. The *x*-axis shows the duration of imbibition and the *y*-axis the percentage of germinated fruits. The germination performance of unprocessed fruits (grey), those processed by a polishing treatment only (orange) as well as those polished and subsequently washed (blue), is shown side by side. Processing reduces the time until accomplishment of 50% germination significantly (*P *< 0.05) for both lots. Each data point represents the mean ± SE of 4 × 50 fruits

Figure 2 shows that the time needed for the completion of germination was very similar for both lots (A and B) when in an unprocessed state (*T*_50%_ mean time for 50% germination of the fruit germination is 151 h for lot A and 157 h for lot B and not significantly different (*P *> 0.05)). The different processing treatments (‘polished’ and ‘polished + washed’) also enhanced the germination speed similarly for both lots (Fig. 2). Polishing had a germination enhancement effect (*T*_50%_ significantly (*P *< 0.05) lower than in unprocessed fruits), suggesting that the removal of pericarp tissue is removing a possible mechanical constraint to germination and/or decreasing the amount of inhibitory compounds due to less pericarp. The combined treatment, polishing and washing, delivered the best results for both lots with a significantly (*P *< 0.05) lower *T*_50%_ than both the unprocessed and polished fruits. This strongly suggests that the restriction by the pericarp tissue is physiochemical in nature and may be caused by the additional removal of inhibitory compounds by the fruit washing.

**Fig. 3:**
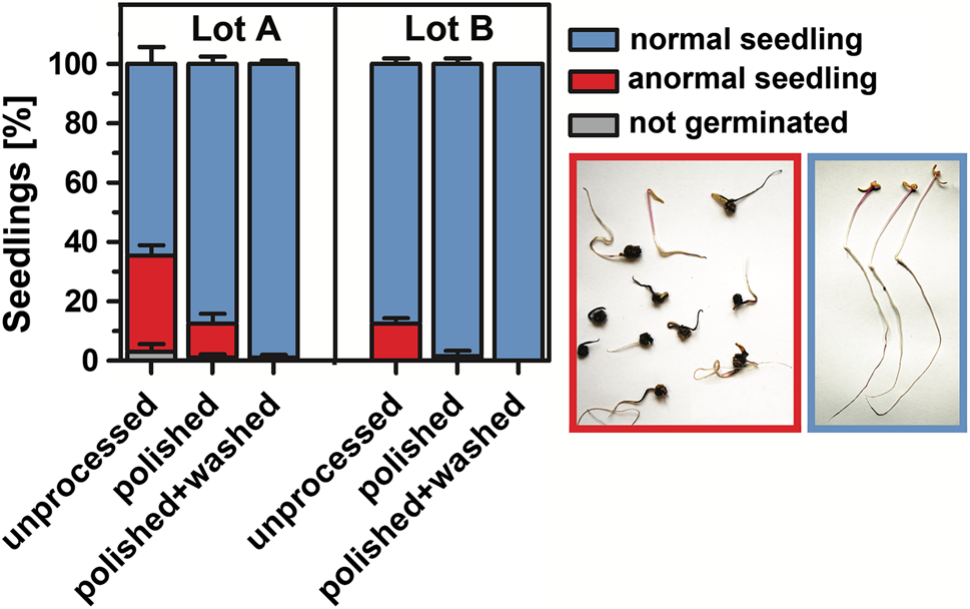
Proportion of normal/anormal sugar beet seedlings of unprocessed, polished, and ‘polished + washed’ fruits. The number of seedlings in each category was recorded after 13 days at 10 °C followed by an additional 3 days at 20 °C. Seedlings emerging from fully processed fruits have generally a normal development and growth physiology of radicle and hypocotyl (blue box). Seedlings emerging from unprocessed fruits produce more (*P *< 0.05) anormal seedlings which are retarded, miss-formed, or darkened in their radicle and hypocotyl (red box). Representative seedlings for the two categories are shown. Each bar represents mean ± SE of 4 replicates of 50 fruits each

Beyond enhancing germination performance, seed quality, and ultimately yield, the most important point of industrial seed- and fruit-processing technologies is to safeguard the development of ‘normal’ seedlings. Normal seedlings are those which have a high probability to grow vigorously enough to be used directly as fresh sprouts (e.g., red beet) or for primary sugar beet crop production in the field (Kockelmann and Meyer 2006; Latorre et al. 2012; Blunk et al. 2017). Figure 3 shows that the appearance of the seedlings was also affected by the different treatments. After an incubation period of 13 days at 10 °C followed by additional 3 days at 20 °C, only unprocessed fruits from lot A had ungerminated fruits (3.0% ± 1.3). In general, anormal seedlings appeared in higher (*P *< 0.05) numbers in lot A than in lot B. The highest proportion (*P *< 0.05) of anormal seedlings were observed in unprocessed fruits (lot A 32.5% ± 1.7 and lot B 12.5% ± 1.0), an effect significantly lessened by either the application of polishing or ‘polishing + washing’. Normal seedlings were observed for almost every fruit after both the treatments (polishing and washing) were applied in combination (Fig. 2).

### Germination inhibition by pericarp wash-water

**Fig. 4:**
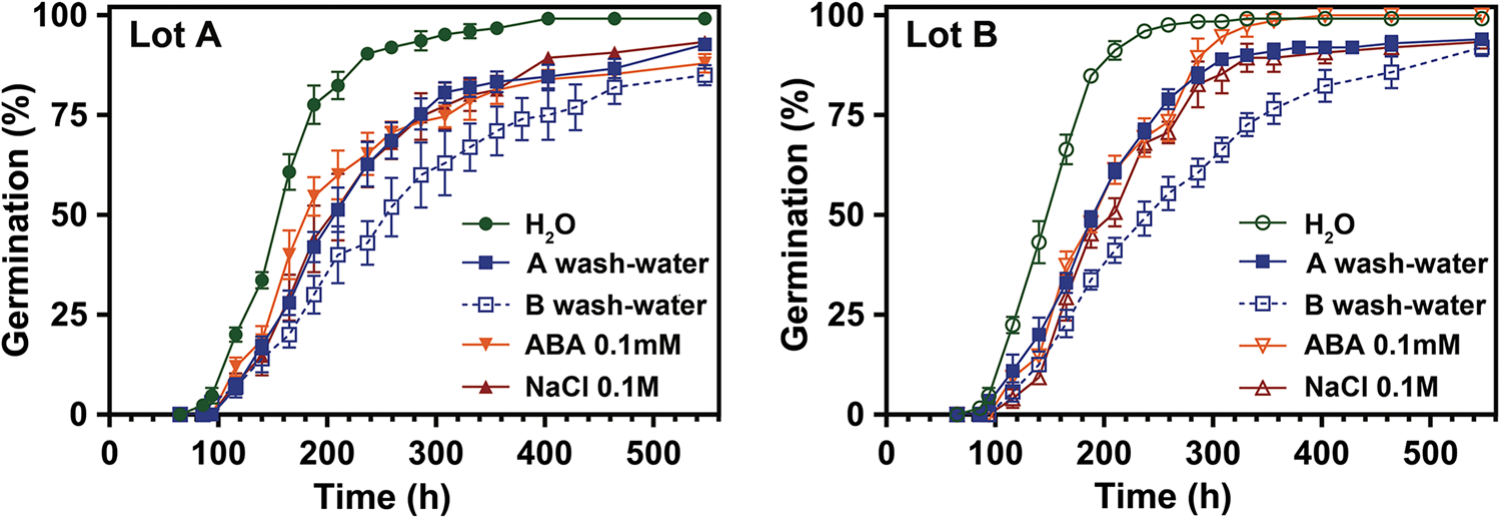
Germination kinetics for sugar beet fruits imbibed with pericarp wash-water, ABA or NaCl at 10 °C in darkness. The percentage of germinated polished and washed fruits was recorded over 550 h of imbibition in water extracts (wash-water) from the pericarps of unprocessed fruits of lot A (in blue, filled) or lot B (in blue, open). Germination performance in the pericarp wash-water solutions was compared against three controls: dH_2_O (in green); 0.1 mM abscisic acid (in orange) and 0.1 M sodium chloride (in red). The *x*-axis shows the duration of imbibition and the *y*-axis shows the percentage of germinated fruits. Each data point represents mean ± SE of at least 3 replicates of 25 fruits each

To establish the negative effect of solvable molecules contained in the pericarp, an experiment was performed to show to what degree water-soluble extracts from crushed pericarps (‘wash-water’) of lot A and lot B can inhibit the germination of both lots. For both lots, fruits that had been polished and washed were imbibed in the pericarp wash-water generated from both lots individually (Fig. 4). These observations were compared with known inhibitory conditions: a hormone treatment (ABA), a salt solution (NaCl), and a control (dH_2_O). Osmolality and conductivity of washwater A (30 ± 1 mOsm/kg H_2_O and 1.9 ± 0.1 mS/cm) and washwater B (55 ± 3 mOsm/kg H_2_O and 4.0 ± 0.1 mS/cm) were measured and compared to for 100 mM NaCl (192 ± 3 mOsm/kg H_2_O and 11.2 ± 0.1 mS/cm), 50 mM NaCl (94 ± 2 mOsm/kg H_2_O and 5.8 ± 0.1 mS/cm), and 25 mM NaCl (46 ± 1 mOsm/kg H_2_O and 2.9 ± 0.1 mS/cm). Imbibition in the pericarp washwater from either lot significantly (*P *< 0.05) slowed down the germination of ‘polished + washed’ fruits in both lots (Fig. 4). The observed inhibitory effects were quantified and compared by calculating the increase in time required to complete 50% germination in the fruit populations. The delay in germination caused by imbibition in washwater derived from unprocessed lot B pericarps was stronger (*P *< 0.05) compared to washwater from unprocessed lot A pericarps (Fig. 4). The delay for 50% germination in lot A ‘polished + washed’ fruits was 52 h for washwater A and 98 h for washwater B, and in lot B fruits, this was 45 h and 96 h, respectively. When compared alongside the germination inhibitory hormone ABA and salt (NaCl), the inhibitory effects of wash-water derived from lot A pericarps corresponded to 100 µM ABA and 100 mM NaCl. These inhibitory solutions and washwater from lot A caused a similar delay in germination for both ‘polished + washed’ lots (for lot A fruits, 50% germination is delayed in washwater A by 52 h, 100 µM ABA by 35 h, and 100 mM NaCl by 53 h and in lot B fruits 45 h, 48 h, and 58 h, respectively). Washwater derived from lot B pericarps showed a much stronger (*P *< 0.05) delay for ‘polished + washed’ fruits of both lots (98 h for lot A fruits and 96 h for lot B fruits) compared to 100 µM ABA and 100 mM NaCl (Fig. 4). Taken together, these findings suggest that unprocessed lot B pericarps contain significantly higher water-soluble inhibitory compounds compared to unprocessed lot A pericarps and that these are effectively removed by the polishing and washing processing. Interestingly, the results of conductivity and osmolality analyses for both washwaters were much lower than those for 100 mM NaCl, rather comparable to values between 25 mM and 50 mM NaCl. This observation indicates that ions are indeed a contributory, but not major factor for the inhibition of germination.

### ABA and ABA–metabolite extraction and quantification

**Fig. 5:**
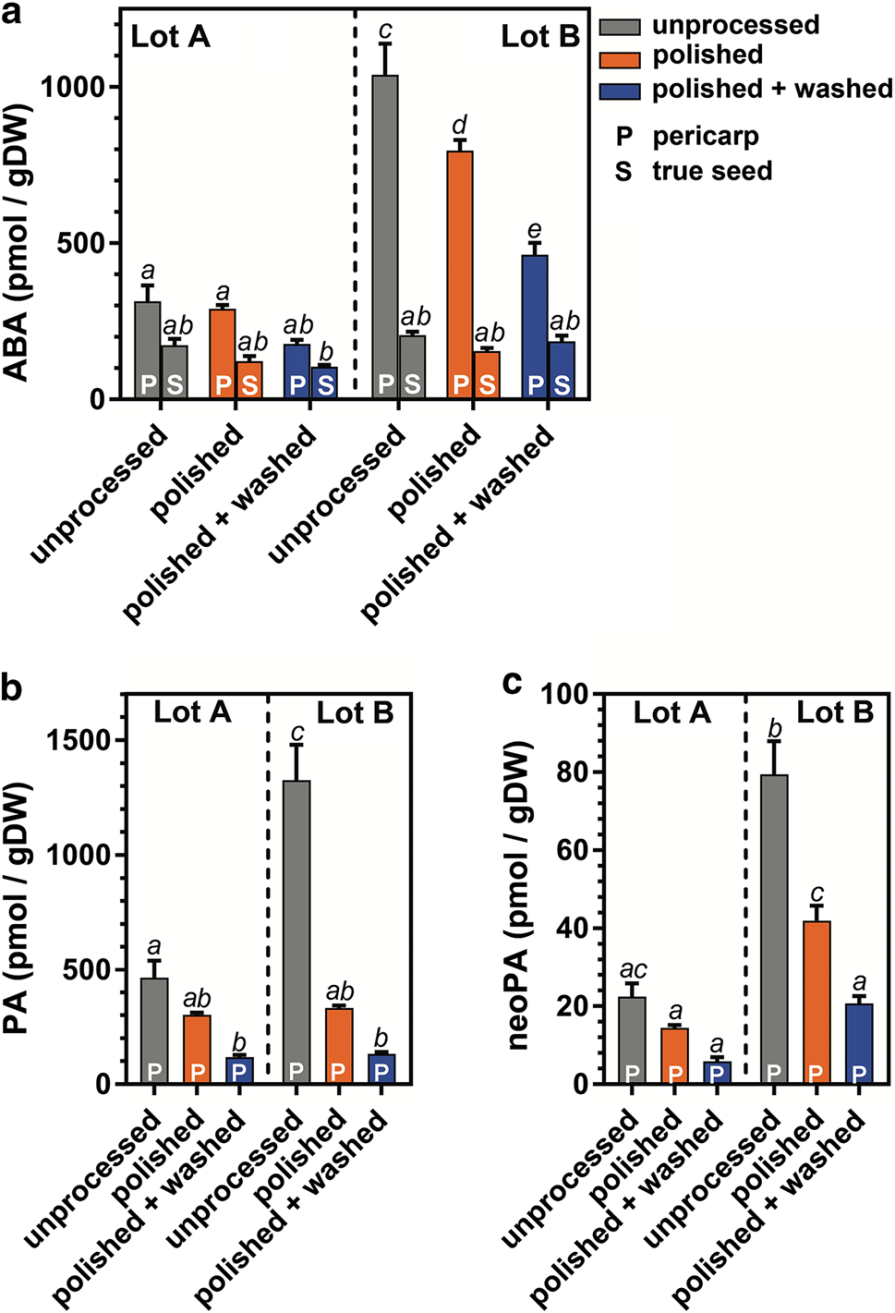
Quantification of the abscisic acid (ABA) and its metabolites phaseic acid (PA) and neophaseic acid (neoPA) in sugar beet fruits. These phytohormone metabolites were quantified in unprocessed (in grey), polished (in orange) and polished + washed (in blue) fruits of lots A and B. Results for the true seed (S) and pericarp (P) is shown separately. Contents of PA and neoPA were very low/below detection limit in true seeds and are not shown. Each bar represents mean ± SE of 5 replicates. Each replicate consists of 50 fruits

To investigate the phytohormone content in pericarps during processing, we analyzed the changes in the contents of ABA and its degradation pathway metabolites for the individual processing steps. The analysis was performed in isolated true seeds and in separated pericarps (including operculums) prepared from ‘unprocessed’, ‘polished’ and ‘polished + washed’ fruits. The ABA contents (Fig. 5a) for most samples are higher in lot B then lot A, with the ABA contents in true seeds being consistently lower than in the pericarp. The ABA contents of unprocessed lot B pericarps (1039 ± 101 pmol/g DW) were ~ 3.3-fold higher compared to unprocessed lot A pericarps (314 ± 52 pmol/g DW). Assessing the effects of the processing on the fruits, the contents of ABA (as well as PA and neoPA) in the pericarps decrease due to the polishing and washing ~ 2.3-fold in lot B (to 463 ± 39 pmol ABA/g DW) and ~ 1.8-fold in lot A (to 178 ± 13 pmol ABA/g DW) pericarps (Fig. 5a). Polishing alone already reduced the pericarp ABA contents of lot B by ~ 1.3-fold (to 796 ± 35 pmol/g DW), but did not significantly affect the lot A contents. The contents of ABA degradation products PA and neoPA (Fig. 5b, c) in pericarps showed very similar trends to those of ABA. In true seeds, contents of the ABA degradation products were very low/below detection limit (data not shown). The degradation products demonstrate that the known ABA inactivation pathways are evident in the pericarp tissue of both lots. The finding that ABA content was more than threefold higher in lot B pericarps compared to the lot A pericarps supports the view that ABA contributes to the higher inhibitory activity of lot B pericarp washwater compared to lot A in the germination assays (Fig. 4). The other ABA metabolites that were screened for were either below detection limits or showed no significant differences between treatments (data not shown).

### Pericarp total solute content osmolality and conductivity

**Fig. 6:**
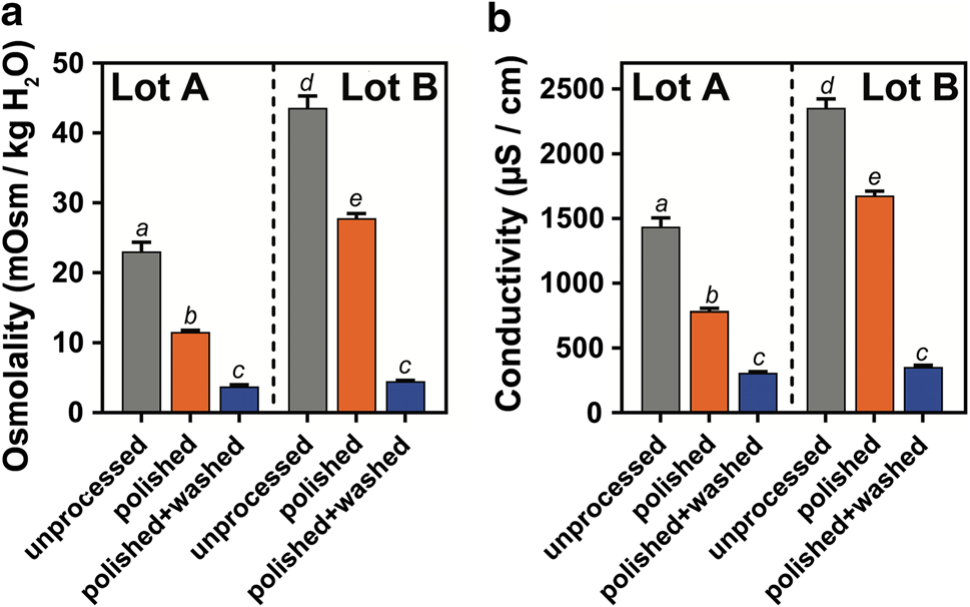
**a** Osmolality and **b** conductivity of sugar beet pericarp wash-water extracts both act as a proxy for salts derived from unprocessed (in grey), polished (in orange) and polished and washed (in blue) pericarps of lots A and B. Unprocessed and polished fruits from lot B have higher values than those from lot A. Washing of polished fruits strongly reduces the values from both lots. Each data point represents mean ± SE of 5 replicates. Each replicate represents a washwater extract from 50 fruits

To examine the ion content in the powdered pericarps of unprocessed, polished and polished + washed fruits, water was used as a solvent and osmolality and conductivity was measured. The osmolality and electrical conductivity can be used as proxy for osmotically active substances including salts (Podlaski and Chrobak 1986; Matthews and Powell 2006). The observed reduction in ions due to polishing and washing are similar for both lots, but the total values are higher for lot B (Fig. 6a, b). When solute content of polished pericarps is compared to unprocessed pericarps, it had a 2.0-fold lower osmolality for lot A and 1.6-fold lower osmolality for lot B, additionally a 1.8-fold lower conductivity for lot A and 1.4-fold lower conductivity for lot B. Interestingly, when extracts from pericarps treated with a combined polishing and washing treatment are compared to extracts from unprocessed pericarps, they have a 6.1-fold lower osmolality for lot A and a 9.7-fold lower osmolality for lot B, as well as a 4.6-fold lower conductivity for lot A and a 6.6-fold lower conductivity for lot B. The differences in osmolality between the pericarp extracts [unprocessed versus polished + washed, pericarp powder in dH_2_O at a ratio of 1:20 (w/w)] is equivalent to a change in NaCl concentration from 13.1 mM to 3.1 mM for lot A and from 23.8 mM to 3.5 mM for lot B. The strongest reduction in osmotically active substances in pericarps was measured after both the treatments (polishing and washing) were applied in combination. In addition, higher osmolality and electrical conductivity of pericarp extracts from lot B compared to lot A support the view that the extracted compounds could contribute to the higher inhibitory activity of lot B pericarp wash-water compared to lot A in the germination assays (Fig. 4).

### Scanning electron microscopy

**Fig. 7:**
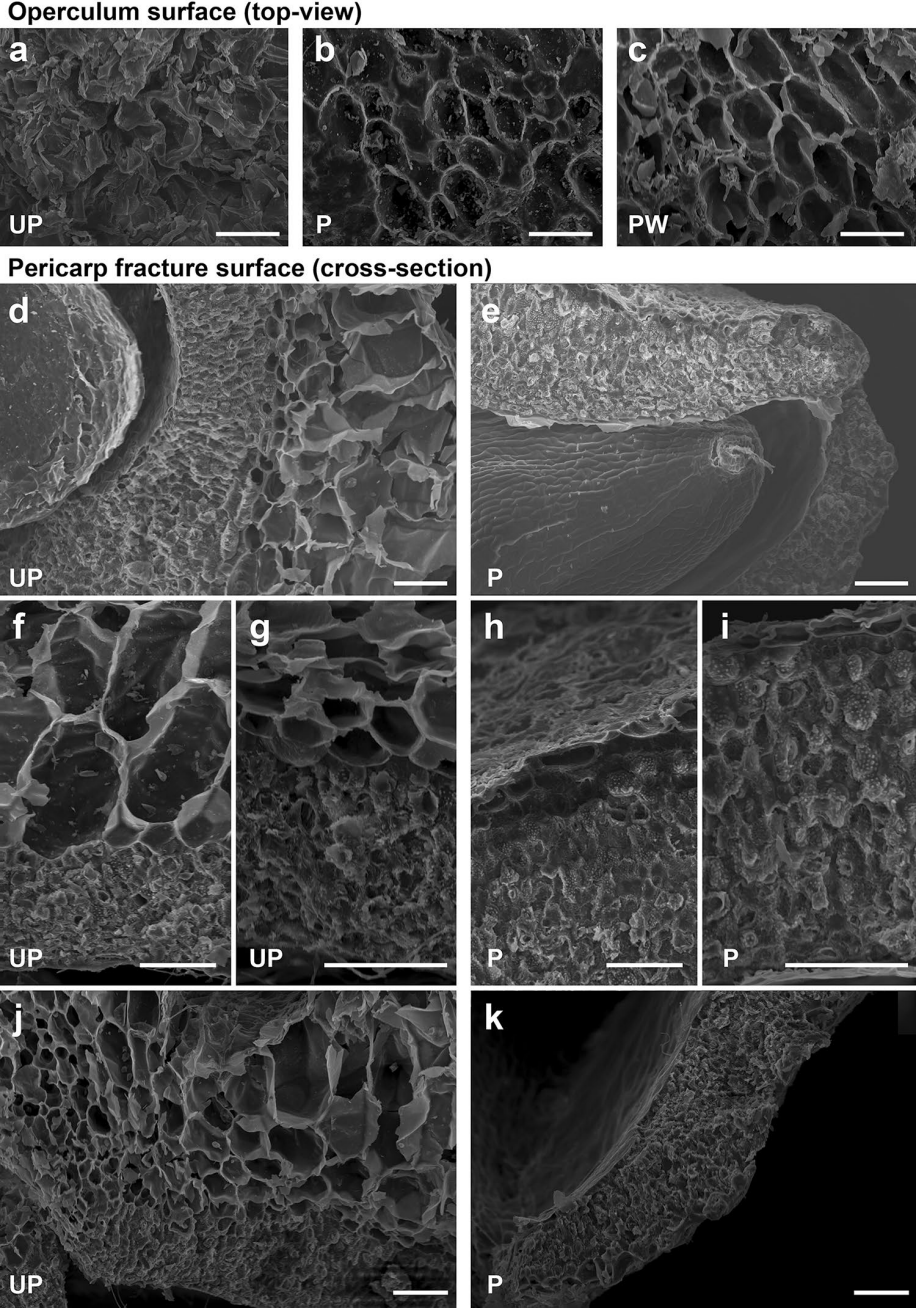
Morphology of the sugar beet pericarp surface and internal structure by scanning electron microscopy (SEM). Images show the operculum surface (**a**–**c**) or fracture surfaces of manually ruptured pericarps (**d**–**k**) of unprocessed (UP) (**a**, **d**, **f**, **g**, **j**), polished (P) (**b**, **e**, **h**, **i**, **k**) and polished and washed (PW) (**c**) fruits of lot B. The scale bar represents 100 µm in each image

Processing treatments affect also the physical aspects of the fruits. Polishing has a large impact on the fruit size and fruit weight (Fig. 1, Table 1). We, therefore, used scanning electron microscopy to gain insight into the morphological changes in the pericarp structure due to both processing steps. Figure 7a–c top view onto the surfaces reveals structural details of the operculum in unprocessed, polished, and polished + washed fruits. The surface view of unprocessed fruits shows the unmodified/original structures of the outer pericarp (Fig. 7a). The surface view of polished fruits (Fig. 7b) is very different, as the outer pericarp layer was largely removed, leaving just the remains of large parenchyma cells on the newly formed surface. Visible also (Fig. 7b), are many particles of various sizes inside the scaffold of the parenchyma cells, most likely cell-wall fragments which were generated by the polishing. The additional washing treatment (Fig. 7c) removes most of these fragments.

Cross-sectional observations on the pericarp tissue of fractured unprocessed fruits (Fig. 7d, f, g, j) show a clear differentiation between two structural layers: an inner layer of thick-walled sclerenchyma cells (‘inner pericarp’) and a porous outer layer of large parenchyma cells (‘outer pericarp’). Those two types of pericarp regions/layers can be distinguished, based on the cell structure, such as size, shape, and cell-wall thickness (Artschwager 1927). In some areas of the pericarp, the distinction between both layers is very clear (Fig. 7d), while occasionally, sub-layers of parenchyma cells can be identified (Fig. 7j), based on the cell size only (bigger in the periphery, smaller towards the centre). When comparing the fracture surface of the inner and outer pericarps, the fracture through the outer pericarp runs through the interior of dead parenchyma cells (revealing the rounded interior of the cell), while the fracture through the inner pericarp runs through the apoplast (Fig. 7f, g, cf. h, i) (most likely between cell walls of adjacent cells), leaving the cell junctions/plasmodesmata visible (under high magnification). We also noted that the organization of layers is similar in the operculum and the pericarp.

The morphology of polished fruits is highly altered when compared to unprocessed fruits (Fig. 7e, k). Fracture surfaces of manually ruptured pericarps show that polishing often removes all but the last or penultimate row of cells in the outer pericarp. The inner pericarp remains unaffected by the polishing (Fig. 7h, i). The smaller sclerenchyma cells form a firm and compact structure within the pericarp. There was no evidence of an effect of washing alone (without a polishing step) on the cell layers and structures in the pericarp and operculum (data not shown).

## Discussion

### The sugar beet fruit pericarp as the target of polishing and washing during the industrial processing to improve quality and germination performance

Processing technologies such as polishing and washing are widely applied to seeds and fruits to enhance their physiochemical properties to provide the best quality product to the food supply chain (Dewar et al. 1998; Taylor et al. 1998; Sliwinska et al. 1999; Sharma et al. 2009; Pedrini et al. 2017; Steinbrecher and Leubner-Metzger 2017). In the dry fruits of *B. vulgaris,* the pericarp (fruit coat) is the clear target of polishing and washing processing treatments. The aim of polishing in general is the reduction of pericarp, thereby removing inhibitors, homogenizing fruits into a usable size fraction and to modify geometry (reducing the star-like structure), thus enabling pelleting for use in conventional drilling machines. In addition, this improves the germination performance for the production of fresh sprouts as salad components (red beet) and primary crop establishment to ensure yield potential via sugar-containing tap root growth (sugar beet) (Morris et al. 1984; Kockelmann and Meyer 2006; Latorre et al. 2012; Tohidloo et al. 2015; Blunk et al. 2017). Despite this importance, the structural and textural changes as well as the underpinning biochemical mechanisms associated with the industrial polishing and washing processes are poorly understood. In some species, the process of dry seeds and fruits to improve germination performance alters the seed or fruit coat morphology to only a minimal degree, in the case of *B. vulgaris*, more drastic alterations to the morphology and biochemistry occur. These are changes, that both we and other authors show, and are required to achieve enhanced physiological quality and germination performance.

We show here that the industrial polishing removed ~ 31 to 35% of the sugar beet fruit outer pericarp tissue and generated a new artificial outer surface with different properties (Figs. 1, 7). During the development and maturation drying of the *B. vulgaris* fruit, two major pericarp tissue layers emerge: A denser inner pericarp layer consisting of thick-walled sclerenchyma cells with many clearly visible plasmodesmata (Fig. 7i), and a more porous outer pericarp layer consisting of large but thin-walled parenchyma cells (Fig. 7f). Our distinction between a morphologically different inner and outer pericarp of beet fruits is consistent with earlier microscopic work (Artschwager 1927; Orzeszko-Rywka and Podlaski 2003; Hermann et al. 2007). The tissues of the inner and outer pericarp are clearly distinguished by distinct cell sizes, forms, and cell walls. Both tissue types are present in the lower pericarp and in the operculum (fruit cap). The operculum is a key feature of the sugar beet fruit, and it is a domed or lid-like structure that covers the true seed with its coiled embryo (Fig. 1). The uptake of both oxygen and water is limited by structures such as the operculum and the basal pore (Chetram and Heydecker 1967; Heydecker et al. 1971; Coumans et al. 1976; Richard et al. 1989; Santos and Pereira 1989). During the late phase of sugar beet germination, the operculum is lifted, and the radicle protrudes through the gap between the lower pericarp and the operculum. Isolated true seeds, which are removed entirely from the pericarp or fruits with the operculum removed, germinate faster in comparison with intact fruits (Coumans et al. 1976; Taylor et al. 2003; Hermann et al. 2007), demonstrating that sugar beet germination is controlled at least partly by the pericarp.

Our electron microscopic investigation demonstrates that polishing removes most of the parenchyma cells of the pericarp, but there is no evidence that sclerenchyma cells were removed (Fig. 7h, i). We, therefore, assume that mechanical removal of the large parenchyma cells in the outer pericarp requires lower shear forces during the polishing process than the sclerenchyma cells of the inner pericarp. Our finding that the removal of the outer porous pericarp is achieved by polishing is consistent with work by others (Orzeszko-Rywka and Podlaski 2003; Tohidloo et al. 2015). However, to uncover, the mechanical differences between the parenchyma and sclerenchyma layers and their response to polishing would require a detailed biomechanical study of the sugar beet pericarp.

The polishing generated an artificial new outer surface with different properties and with adhering particles of various sizes inside the scaffold of the parenchyma cells (Fig. 7b). Washing of polished sugar beet fruits removed the adhering particles (Fig. 7c) and washing also removed the requirement to position the fruit with the operculum downwards onto germination plates (Santos and Pereira 1989). Both polishing and washing generate altered new outer surface structures which are important for the improved adhesion of coating and pelleting materials (Duan and Burris 1997; Taylor et al. 1998; Kockelmann and Meyer 2006; Pedrini et al. 2017). The proper integration of the polishing and washing steps into the entire processing scheme is, therefore, important.

### Polishing and washing both contributed to the removal of pericarp-localised chemical inhibitors of sugar beet germination

The removal by polishing and washing of two groups of germination inhibiting chemical compounds localised in the pericarp of sugar beet fruits was studied in lot A and lot B. The most important finding is that the combined polishing and washing treatment resulted in a significant improvement of the germination performance in both lots (Fig. 1) due to a combined removal of mechanical (see preceding section) and inhibitor constraints. Chemical inhibitors in the sugar beet and red beet pericarp are known to be inorganic ions including various cations (mainly Na^+^ and K^+^) and anions (mainly Cl^−^ and oxalate) (Junttila 1976; Morris et al. 1984; Podlaski and Chrobak 1986). In agreement with their role as inorganic inhibitors, NaCl solutions of at least 100–300 mM were required to inhibit sugar beet fruit germination (Fig. 4 this work; and Junttila 1976; Morris et al. 1984; Podlaski and Chrobak 1986). Electrical conductivity of pericarp washwater was proposed to be a useful method to monitor the efficacy of industrial washing procedures (Podlaski and Chrobak 1986; Orzeszko-Rywka and Podlaski 2003). These authors found that pericarp wash-water inhibited sugar beet germination if the conductivity value was above ~ 10 mS/cm, which is slightly less than the 100 mM NaCl solution used in our germination experiments (11.2 mS/cm; Fig. 4). The pericarp washwater of our unprocessed sugar beet lots A and B had conductivity values of about 1.9 mS/cm and 4.0 mS/cm, respectively, but this clearly inhibited the germination of both lots (Fig. 4). We conclude from this that the salts in pericarp washwater from lots A and B alone cannot confer the inhibition, though they most likely contribute. Further to this, we found a clear decrease in the conductivity during polishing and washing by at least 4.6-fold, which is well below the NaCl concentration which inhibits sugar beet germination and confirms that the combined polishing and washing reduced the contents of inorganic salts in the pericarp to very low values to improve the germination performance. We also show that measuring the osmolality of the pericarp washwater is a suitable alternative to measuring the electrical conductivity. Earlier work also demonstrated that diverse phenolic compounds isolated from either red beet or sugar beet pericarps inhibited the seed germination and seedling growth of target species such as lettuce, cress (Mitchell and Tolbert 1968; Battle and Whittington 1969; Chiji et al. 1980). These compounds which accumulate in the pericarp including protocatechuic, *p*-hydroxybenzoic, vanillic, ferulic, *p*-coumaric, and salicylic acid are also found in similar material by Peukert et al. (2016). Taken together, our polishing and washing treatment effectively reduced the contents of organic and inorganic compounds in the sugar beet pericarp, but these germination inhibitors are not sufficient to explain the fact that pericarp washwater from lot B had a stronger inhibitory effect on the germination when compared to pericarp washwater from lot A (Fig. 4).

The second group of chemical inhibitors in the sugar beet pericarp we studied were plant hormones. Earlier work has demonstrated (Battle and Whittington 1969; Hermann et al. 2007) that the pericarp tissue contains high levels of ABA in the micromolar range. Above 1 µmol ABA per gram of dry pericarp was reported by Hermann et al. (2007) and in our work here for unprocessed fruits of lot B, whereas lot A had lower values of ~ 0.3 µmol ABA per gram (Fig. 5a). We propose that the > 3-fold higher ABA contents of lot B compared to lot A are the major reason for the higher inhibitory effect of pericarp wash-water from lot B when compared to lot A (Fig. 4). Polishing and washing effectively decreased the ABA content of both lots and were associated with washing out ABA from the pericarp, combined with biochemical ABA inactivation via the known 8′ and 9′ enzymatic hydroxylation pathways (Grappin et al. 2000; Ali-Rachedi et al. 2004; Nambara et al. 2010) as evident from the metabolites PA and neoPA (Fig. 5b, c). Interestingly, with regard to the total pericarp weight, a similar amount of ABA was localised in the inner pericarp (53% in lot B and 60% in lot A) when compared to the outer pericarp. It, therefore, requires the combined polishing and washing treatment to reduce the ABA contents to a low value. Treatment with 100 µM ABA effectively inhibited the germination of lot B and A (this work) and of a polished lot used by Hermann et al. (2007). In conclusion, while the different ABA content in the inner pericarp layer explains the difference between lot B and lot A in germination speed and pericarp wash-water inhibitory activity, it is the combined removal of various groups of inhibitors (ABA, phenolic compounds, salts, and others) by the polishing and washing process which helps achieve the full germination potential of each individual fruit.

### The production of usable beet seedlings is dependent upon the quality of the fruit polishing and washing procedures

The polishing and washing of sugar beet fruits both increased the percentage of normal (usable) seedlings. For a technology to be applied in the seed industry ideally, no anormal or a very low prevalence of anormal seedlings is crucial (Taylor et al. 1998; Kockelmann and Meyer 2006; Pedrini et al. 2017). Tohidloo et al. (2015) also reported a reduction of anormal seedlings after sugar beet fruit washing, but in contrast to our findings, their polishing increased the number of anormal seedlings, potentially via mechanical damage of the embryo during the polishing. This demonstrates that an optimised engineering of the polishing treatment is crucial for the best output of usable seedlings. Finally, post-polishing and washing, and sugar beet fruits are primed, film coated, and pelleted to provide the highest quality of commercial seed ready for market.

## Conclusions

This research demonstrated that the individual processing treatments, polishing and washing, have a positive effect on the germination performance and seedling phenotype; this positive effect is stronger when both treatments are combined. The biochemical mechanism underpinning these improvements in seed quality includes a substantial reduction of germination inhibitors found in the pericarp. The ABA, ABA metabolites, and ion content of the pericarp greatly decreased, particularly when the two processing treatments were combined. The shear forces generated during the polishing process removed the outer pericarp tissue (parenchyma), whereas the denser tissue (sclerenchyma) was preserved. Processing treatments such as polishing and washing, especially in combination, are a useful tool to improve the quality and germination performance of commercial seed.

### *Author contribution statement*

MI, TS, JEH, and GL-M planned and designed the research; MI and VT performed experiments; MI, JEH, TS, UF, and JM provided material or analytical tools; MI, VT, MS, TS, JEH, and GL-M analyzed and interpreted the data; JEH, MI, TS, and GL-M wrote the manuscript with contributions of all authors.

## Data Availability

All data presented or analyzed in this published article are available online through figshare 10.17637/rh.8208566.

## Abbreviations

PA: Phaseic acid
SEM: Scanning electron microscopy
